# Development and evaluation of a two-tube multiplex real-time PCR assay for differential detection of Monkeypox virus clades Ia, Ib, IIa, and IIb

**DOI:** 10.3389/fmicb.2026.1907267

**Published:** 2026-09-11

**Authors:** Xiangyu Zhu, Ning Shi, Yong Liu, Jiachen Li, Jiaxin Tian, Bocheng Liu, Xuerui Wang

**Affiliations:** 1 Department of Clinical Laboratory, The Second Hospital of Jilin University, Changchun, Jilin, China; 2 The Second Department of Infectious Disease Prevention and Control, Center for Disease Control and Prevention in Northern Theater Command, Shenyang, Liaoning, China; 3 State Key Laboratory for Diagnosis and Treatment of Severe Zoonotic Infectious Diseases, Key Laboratory for Zoonosis Research of the Ministry of Education, Institute of Zoonosis, and College of Veterinary Medicine, Jilin University, Changchun, Jilin, China

**Keywords:** diagnosis, Monkeypox virus, MPXV clades, multiplex qPCR, rapid detection

## Abstract

Monkeypox virus (MPXV) has caused two Public Health Emergencies of International Concern since 2022, with co-circulation of distinct clades (Ia, Ib, IIa, IIb) that differ in virulence and transmission patterns. Rapid discrimination of these clades is critical for outbreak control and clinical management. Here, we developed and validated a two-tube multiplex real-time quantitative PCR (qPCR) assay targeting the H3L gene, designed to first distinguish clade I from clade II, followed by subtyping into clades Ia, Ib, IIa, and IIb in separate reactions. The assay demonstrated high sensitivity, with detection limits of 100 copies/reaction for clade Ia, 10 copies/reaction for clade Ib, 10 copies/reaction for clade IIa, and 1 copy/reaction for clade IIb using standard plasmids. Excellent specificity was confirmed by the absence of cross-reactivity with nucleic acids from other human pathogens, including vaccinia virus Tiantan strain (VTT), human adenovirus (HAdV), getah virus (GETV), chikungunya virus (CHIKV), Japanese encephalitis virus (JEV), dengue virus (DENV), influenza A virus (IAV), influenza B virus (IBV), and severe acute respiratory syndrome coronavirus 2 (SARS-CoV-2). The assay exhibited high repeatability, with intra-assay coefficients of variation (CVs) ranging from 0.09 to 1.88% and inter-assay CVs from 0.23 to 2.68%. Clinical evaluation using 48 skin samples from infected crab-eating monkeys or SCID mice showed 100% concordance with singleplex qPCR for clade IIb detection. For clades Ia, Ib, and IIa, clinical validation was not feasible due to sample unavailability, and their performance was analytically confirmed using recombinant plasmids. Importantly, the clade assignment of the 48 animal specimens was not confirmed by sequencing, which represents a limitation of the current study. Furthermore, these specimens were derived from animal models rather than human patients; thus, the results represent preclinical validation, and formal clinical validation in human specimens from endemic regions is warranted. This two-tube multiplex qPCR assay provides a rapid, sensitive, and reliable tool for the analytical discrimination of all four major MPXV clades, with preliminary clinical validation for clade IIb. Its ability to distinguish emerging clades makes it a valuable asset for clinical diagnosis, epidemiological surveillance, and future outbreak preparedness, although further clinical validation in endemic regions is required.

## 1 Introduction

In 1980, the World Health Organization (WHO) declared Variola virus eradicated (Behbehani, 1983); however, *orthopoxviruses* remain a public health concern due to their zoonotic potential and declining population-level immunity following the cessation of smallpox vaccination. Beyond smallpox, several other members of this genus have the potential to infect humans, including monkeypox, cowpox, and other zoonotic *orthopoxviruses* transmitted by rodents (Doty et al., 2019; Gigante et al., 2019; Springer et al., 2017; Vora et al., 2015).

Among these, MPXV has emerged as the most significant *orthopoxvirus* threatening humans since the eradication of smallpox. MPXV is a double-stranded DNA virus (approximately 197 kb) belonging to the genus *orthopoxvirus*, family *Poxviridae* (ICTV, 2024). First isolated in 1958 (von Magnus et al., 1959), the first human case was documented in 1970 in the Democratic Republic of the Congo (Al-Musa et al., 2022). Between 1997 and 2020, recurrent outbreaks in Central and West Africa resulted in over 5,000 cumulative cases.

The epidemiological landscape changed dramatically in May 2022, with cases reported in multiple non-endemic countries, leading to a global outbreak (Endo et al., 2022). On July 23, 2022, WHO declared this a Public Health Emergency of International Concern (PHEIC) (WHO, 2022). In August 2022, WHO introduced a revised nomenclature, classifying MPXV into two major clades: clade I (formerly the Congo Basin clade) and clade II (formerly the West African clade) (WHO, 2022), with clade II further subdivided into IIa and IIb (Happi et al., 2022). Subsequently, in April 2024, clade I was subdivided into Ia and Ib following identification of genetically distinct variants (Soleimani and Motamed, 2024). By August 2024, the resurgence of mpox, particularly the rapid spread of clade Ib, prompted WHO to declare a PHEIC once again (WHO, 2024a). As of December 31, 2025, over 170,000 confirmed cases and nearly 500 deaths have been reported across 133 member states (WHO, 2026).

These clades exhibit marked differences in clinical severity and transmissibility. Clade I is associated with higher virulence (10–11% case fatality rate), whereas clade II generally causes milder disease (Andrei and Snoeck, 2023; Bunge et al., 2022). Within clade I, the recently emerged clade Ib has demonstrated enhanced transmissibility, while clade Ia remains highly endemic in the Democratic Republic of Congo. Clade IIb has established sustained transmission globally (WHO Fourth Meeting of the International Health Regulations, 2005). This co-circulation of differentially pathogenic clades underscores the need for diagnostic tools capable of clade discrimination.

Real-time quantitative qPCR (qPCR) stands as the gold standard for MPXV nucleic acid detection (WHO, 2024b). Several multiplex qPCR-based MPXV detection kits have received regulatory approval globally, primarily targeting the F3L gene (BioGerm, 2024; GENE, 2024; NMPA, 2025; Sansure, 2024a,b). WHO guidelines recommend prioritizing the collection of specimens from skin lesions (rash, swabs, or exudates); in the absence of skin lesions, oropharyngeal or anal swabs should be collected, but blood samples are not recommended for testing (Cabanillas et al., 2024; Fan et al., 2025; Palich et al., 2023; Veintimilla et al., 2022; Yigci et al., 2025). Numerous in-house assays have also been reported, targeting various MPXV genes including N3R (Kulesh et al., 2004), B6R (Li et al., 2006), B7R (Shchelkunov et al., 2011), E9L (Li et al., 2006), and others (Edghill-Smith et al., 2005; Fan et al., 2025; Kurosawa et al., 2025; Li et al., 2010; Lin et al., 2024; Maksyutov et al., 2016). However, these assays were largely developed prior to the recent diversification of MPXV clades and lack the capability to differentiate between all four currently circulating lineages (Ia, Ib, IIa, IIb).

Here, we report the development and validation of a two-tube multiplex qPCR assay targeting the IMV heparin-binding surface protein (H3L) gene of MPXV. The H3L gene was selected due to its relative conservation across orthopoxviruses while harboring lineage-specific single nucleotide polymorphisms (SNPs) suitable for clade discrimination. Here, we report the analytical sensitivity, specificity, repeatability, and preliminary clinical evaluation of this two-tube assay designed to distinguish clades Ia, Ib, IIa, and IIb.

## 2 Materials and methods

### 2.1 Viral nucleic acid

The cDNA/DNA of vaccinia virus Tiantan strain (VTT), human adenovirus (HAdV), getah virus (GETV), chikungunya virus (CHIKV), Japanese encephalitis virus (JEV), dengue virus (DENV), influenza A virus (IAV), influenza B virus (IBV) and severe acute respiratory syndrome coronavirus 2 (SARS-CoV-2) were stored at –80°C. The nucleic acids of the MPXV (clade IIb, GenBank accession number: PP778666.1) samples were obtained from dry swabs or crusts taken from the skin lesions of crab-eating monkeys or SCID mice. Multiplex qPCR experiments were performed in a biological safety cabinet under BSL-2 conditions.

### 2.2 Design of multiplex qPCR primers and probes

**FIGURE 1:**
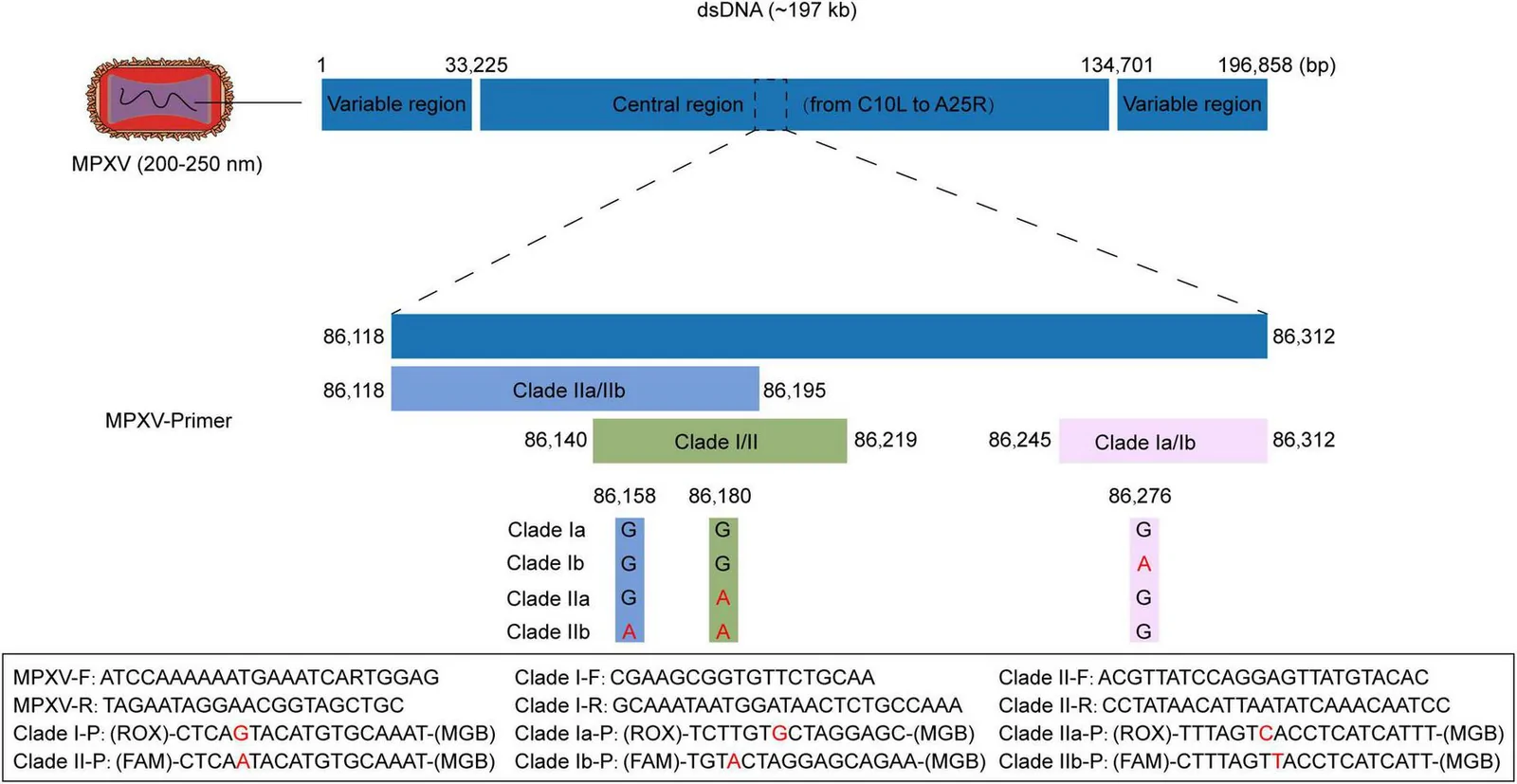
Scheme of fragments used for designing primer/probe sets to clade Ia, Ib, IIa, and IIb, respectively. The red indicates mutation.

A search was conducted of 7,581 MPXV sequences from the GISAID^1^ and NCBI^2^ databases on September 1, 2025. We selected 220 high-quality MPXV sequences for analysis. These sequences were chosen through balanced screening based on three criteria: (i) collection date (spanning from 1960 to 2025 to capture temporal diversity), (ii) geographic location (covering endemic regions in Central and West Africa, as well as non-endemic regions affected by the 2022–2025 global outbreaks), and (iii) genotyping conditions (ensuring proportional representation of clades Ia, Ib, IIa, and IIb based on their relative prevalence). The selected sequences included 60 from clade Ia, 72 from clade Ib, 20 from clade IIa (only two of which were available on GISAID as of September 11, 2024; the remaining 18 were downloaded from NCBI), and 68 from clade IIb. To ensure the robustness of primer and probe design, we performed multiple sequence alignment using DNASTAR software (version 7.0) and calculated the conservation percentage and SNP frequency at each target position. Only regions with > 95% conservation within each clade and containing clade-specific SNPs were selected for primer and probe design. Primers and probes were subsequently designed using Primer Premier 5 software (Premier, Canada) based on these conserved regions. TaqMan probes for clade I, II, clade Ia, Ib, IIa and IIb were labeled with ROX and FAM at the 5′-end, respectively, with MGB used as 3′-end quenchers. An *in silico* specificity analysis was performed by BLAST searching all primer and probe sequences against the NCBI nucleotide database to confirm their uniqueness to target MPXV clades. The sequences of the primers and probes designed in this study are listed in Figure 1 and were synthesized by Sangon Biotech (Shanghai) Co., Ltd.

### 2.3 Preparation of recombinant plasmids

The target genes of clade Ia, Ib, IIa, and IIb were respectively cloned into vector pUC57 for recombinant plasmids (Sangon Biotech (Shanghai) Co., Ltd). The plasmid DNA was quantified using a NanoDrop 2000 (Thermo Scientific, United States). The resulting DNA concentrations were converted to genome copy values using the formula: y (copies/μL) = (6.02 × 10^23^) × (x(ng/μL) × 10^–9^ DNA)/(DNA length × 660). The plasmids were 10-fold diluted from 10^7^ to 10^1^ copies/μL and stored at –20 °C.

y: DNA copy number. x: plasmid concentration (ng/μL).

### 2.4 Establishment of standard curves for multiplex qPCR

All multiplex qPCR assays were performed using a CFX96 Real-Time PCR Detection System (Bio-Rad, United States). Following repeated experimental trials, the reaction conditions for the multiplex qPCR assay were determined as follows: 12.5 μL of Premix EX Taq 2 × (Takara, Japan), 0.5 μL each of Forward and Reverse Primers (final concentration: 0.2 μM each), 0.5 μL each of Probe (final concentration: 0.2 μM each), 2.0 μL of DNA template, and nuclease-free water to a final volume of 25.0 μL. Specifically, Panel 1 contains two probes: Clade I-specific probe (FAM-labeled, 0.2 μM) and Clade II-specific probe (ROX-labeled, 0.2 μM). Panel 2 contains four probes: Clade Ia-specific (FAM, 0.2 μM), Clade Ib-specific (ROX, 0.2 μM), Clade IIa-specific (FAM, 0.2 μM), and Clade IIb-specific (ROX, 0.2 μM). Fluorescence signals were automatically collected at the end of each amplification cycle.

**FIGURE 2:**
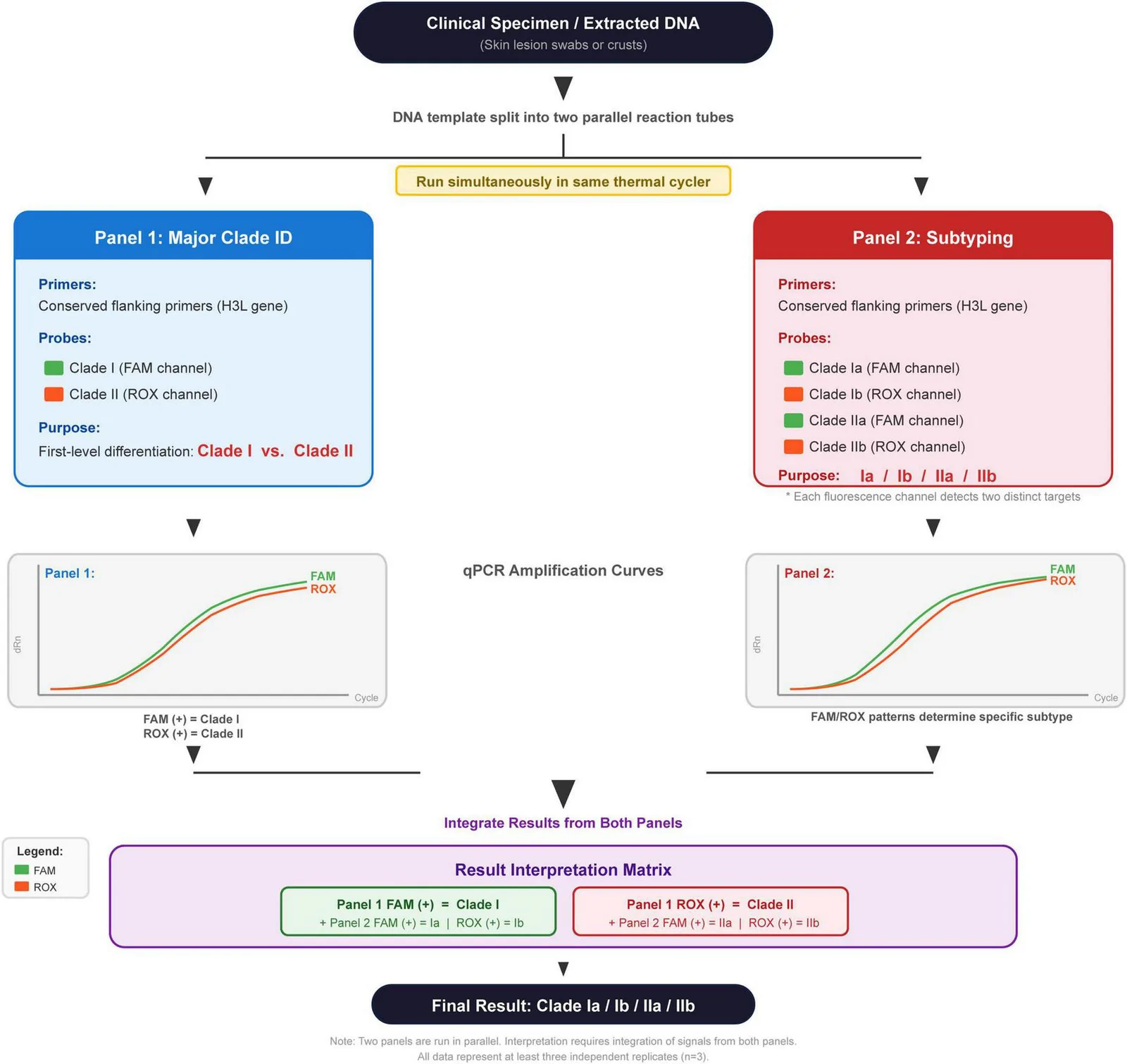
Schematic workflow of the two-tube multiplex qPCR assay. Panel 1 distinguishes clade I from clade II, and Panel 2 subtypes clade I into Ia/Ib and clade II into IIa/IIb. Panels 1 and 2 are run in parallel from the same DNA extract; final clade assignment requires integration of signals from both panels. All data represents at least three independent replicates (*n* = 3).

The assay was designed as a two-tube system (Panel 1 and Panel 2) that are run in parallel from the same DNA extract, with results interpreted by integrating signals from both panels. As shown in Table 1, Panel 1 contains primers and probes targeting the conserved regions of clade I (FAM channel) and clade II (ROX channel), enabling the first-level differentiation of MPXV into major clade I or clade II. Panel 2 contains probes for subtyping: clade Ia (FAM), clade Ib (ROX), clade IIa (FAM), and clade IIb (ROX), with the primers designed to amplify a conserved flanking region common to all four subtypes. Critically, because Panel 2 uses the FAM channel to detect both clades Ia and IIa and the ROX channel to detect both clades Ib and IIb, the Panel 2 signal alone cannot distinguish between clades Ia and IIa, or between Ib and IIb. This distinction requires integration with the Panel 1 result—a two-step decoding strategy that enables four-clade discrimination using only two fluorescence channels. Each tube contained one set of primers and two sets of probes, allowing for two distinct qPCR reactions to be performed simultaneously. A schematic workflow of the two-tube detection system is illustrated in Figure 2.

**TABLE 1:** Composition and target assignment of the two-tube multiplex qPCR system.

Panel	Target clade	Primer set	Probe	Fluorophore	Purpose
Panel 1	Clade I	Conserved flanking primers	Clade I-specific probe	FAM	First-level: distinguishing Clade I
Panel 1	Clade II	Conserved flanking primers	Clade II-specific probe	ROX	First-level: distinguishing Clade II
Panel 2	Clade Ia	Conserved flanking primers	Clade Ia-specific probe	FAM	Subtyping within Clade I
Panel 2	Clade Ib	Conserved flanking primers	Clade Ib-specific probe	ROX	Subtyping within Clade I
Panel 2	Clade IIa	Conserved flanking primers	Clade IIa-specific probe	FAM	Subtyping within Clade II
Panel 2	Clade IIb	Conserved flanking primers	Clade IIb-specific probe	ROX	Subtyping within Clade II

Based on the optimized reaction conditions and thermal cycling protocol, three replicates of plasmid standard mixtures (ranging from 10^7^ to 10^1^ copies/μL) were examined using the multiplex qPCR assay to generate standard curves. These standard curves were derived from linear regression analysis between cycle threshold (Ct) values and the logarithm of plasmid viral load. Each sample was tested in triplicate in an experiment.

### 2.5 Specificity analysis of multiplex qPCR

To evaluate the specificity of the developed qPCR assay, nucleic acid samples of VTT, HAdV, GETV, CHIKV, JEV, DENV, IAV, IBV and SARS-CoV-2 were used as templates. Plasmid standard mixtures at a concentration of 10^6^ copies/μL were used as positive controls, and nuclear free water was included as a negative control. Each sample was tested in triplicate.

### 2.6 Sensitivity analysis of multiplex qPCR

To provide a preliminary estimate of the limit of detection (LOD), 10-fold serial dilutions of the plasmid standard mixtures, ranging from 10^7^ to 10^1^ copies/μL, were used as templates for amplification. Each dilution was tested in triplicate. The LOD was defined as the lowest concentration at which all three replicates yielded detectable amplification signals. This preliminary LOD provides a reliable estimate of the assay’s analytical sensitivity within the clinically relevant concentration range. Formal LOD validation with finer concentration gradients and ≥ 20 replicates per concentration is planned for future work.

### 2.7 Repeatability analysis of multiplex qPCR

The repeatability of the qPCR assay was evaluated by calculating the coefficients of variation (CV) for both intra-assay and inter-assay. Plasmid standard mixtures of 10^6^ and 10^4^ copies/μL were used as templates. For intra-assay repeatability, each template was tested in triplicate within a single experimental run. For inter-assay repeatability, each template was analyzed in three independent assays performed on separate days.

### 2.8 Clinical sample detection of multiplex qPCR

A total of 48 nucleic acids from positive specimens were collected from the skin lesions of crab-eating monkeys or SCID mice, including 28 samples from crab-eating monkeys and 20 samples from SCID mice. The extracted DNA was stored at –80 °C until use.

## 3 Results

### 3.1 Principles for distinguishing between the clades Ia, Ib, IIa, and IIb

To elucidate the design strategies for lineage-specific probes, an analysis was conducted of the evolutionary characteristics of MPXV clades, and a summary was made of the single-nucleotide polymorphism (SNP) sites that define its various lineages. It has been established that each of these clades possesses unique SNP sites. According to the sites, probes were designed and labeled with the FAM and ROX fluorophores, respectively. These probes enable the rapid detection of several major clades (clades Ia, Ib, IIa, and IIb). Specifically, we developed probes targeting the unique sites of clade I/II, clade Ia/Ib, and clade IIa/IIb for lineage-specific detection (Figure 1).

### 3.2 Establishment of multiplex qPCR

**FIGURE 3:**
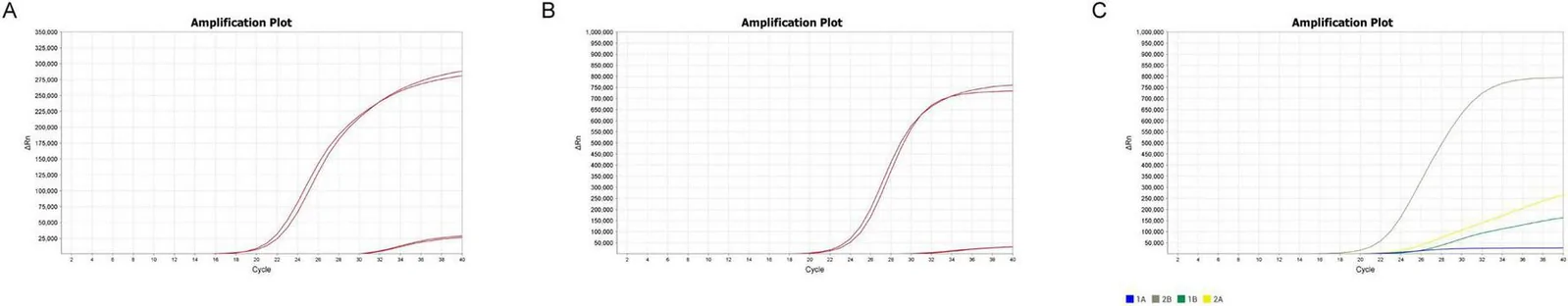
Establishment of multiplex qPCR. The fluorescence signals of FAM and ROX for the clade I **(A)**, II **(B)** and clade Ia, Ib, IIa, and IIb **(C)** were monitored via multiplex qPCR. No fluorescent signal was detected from the negative control (nuclear free water, not shown in the diagram). All data represents at least three independent replicates (*n* = 3).

10^4^ copies/μL of plasmid standard mixtures were analyzed using the optimized multiplex assay. The results demonstrated that the multiplex qPCR assay successfully detected all target genes of the four clades based on the amplification curves (Figures 3A–C). These results indicated that the multiplex qPCR assay was successfully established and functioned reliably. As illustrated in Figure 2 and Table 2, final clade assignment requires integration of signals from both Panel 1 and Panel 2.

**TABLE 2:** Result interpretation criteria for the two-tube multiplex qPCR assay.

Part A: Signal interpretation by panel and channel			
Panel	Channel	Positive	Negative
Panel 1	FAM	Clade I detected	Clade I not detected
Panel 1	ROX	Clade II detected	Clade II not detected
Panel 2	FAM	Clade Ia or IIa detected	Clade Ia or IIa not detected
Panel 2	ROX	Clade Ib or IIb detected	Clade Ib or IIb not detected
**Part B: Final clade assignment (integration of Panel 1 and Panel 2)**			
**Panel 1 Signal**	**Panel 2 Signal**	**Final Interpretation**	**Action**
FAM (+)	FAM (+)	Clade Ia	Report as Clade Ia
FAM (+)	ROX (+)	Clade Ib	Report as Clade Ib
ROX (+)	FAM (+)	Clade IIa	Report as Clade IIa
ROX (+)	ROX (+)	Clade IIb	Report as Clade IIb
FAM (+) + ROX (+)	-	Mixed clade I/II detected	Report as “Mixed MPXV infection detected”; sequencing recommended
FAM (+)	FAM (+) + ROX (+)	Clade I with possible mixed infection	Repeat; if reproducible, sequencing recommended
ROX (+)	FAM (+) + ROX (+)	Clade II with possible mixed infection	Repeat; if reproducible, sequencing recommended
No signal	No signal	Negative	Report as MPXV not detected

### 3.3 Standard curves and analytical sensitivity of multiplex qPCR

**FIGURE 4:**
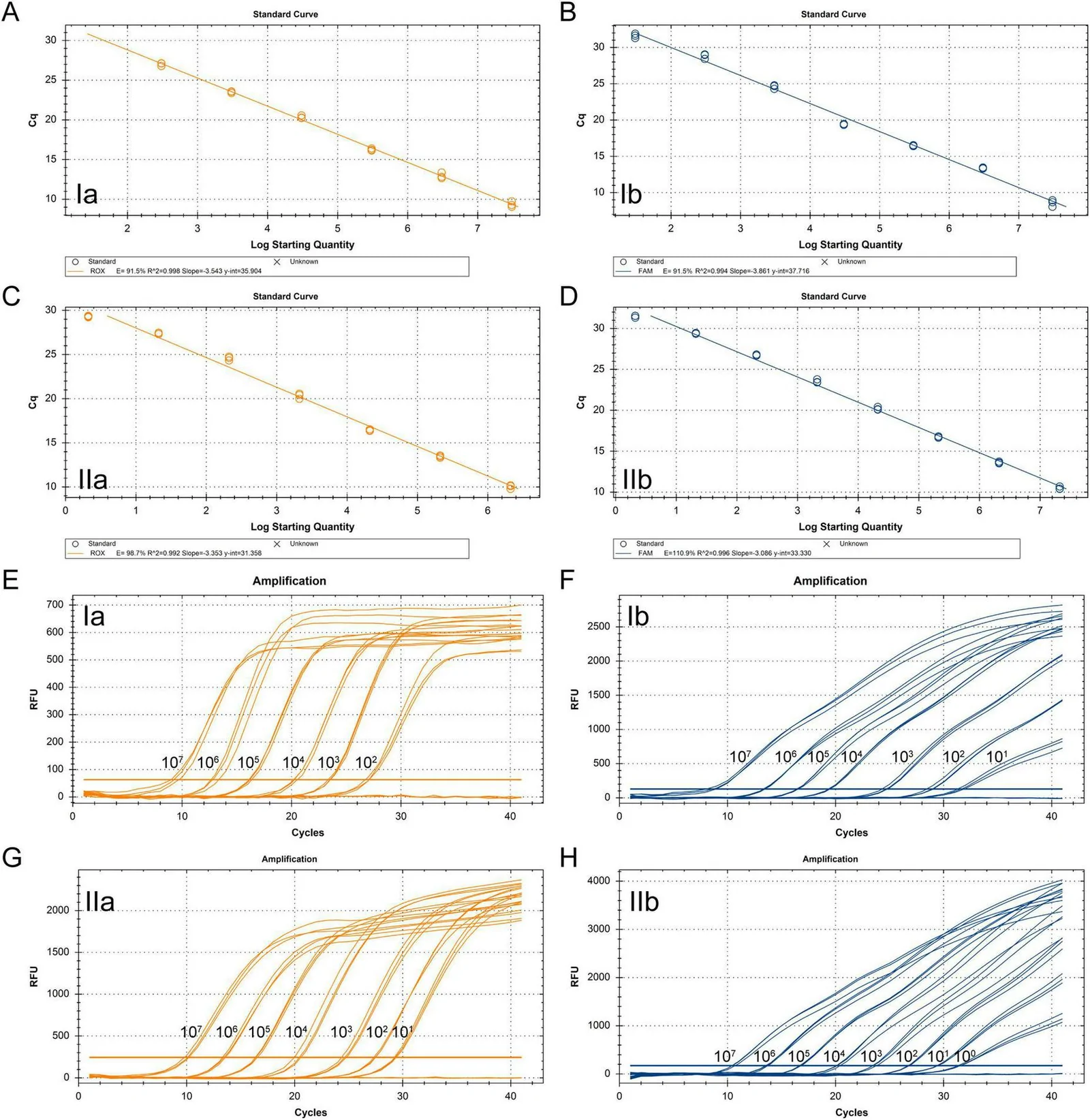
Standard curves and sensitivity of multiplex qPCR. **(A–D)** The standard curves showed an excellent linear relationship (*R*^2^ > 0.990) between the concentration of the standard plasmid and the corresponding Ct values. **(E–H)** The limits of detection were 100 copies/reaction for clade Ia, 10 copies/reaction for clade Ib and clade IIa, and 1 copy/reaction for clade IIb. All data represents at least three independent replicates (*n* = 3).

The construction of standard curves for multiplex qPCR assays is achieved by plotting the cycling threshold (Ct) value against the plasmid viral load (copies/μL). The standard curves exhibited excellent correlation coefficients (R^2^) and amplification efficiency (Eff%) for each clades (Figures 4A–D), with clade Ia (*R*^2^ = 0.998; Eff% = 91.5%), clade Ib (*R*^2^ = 0.994; Eff% = 91.5%), clade IIa (*R*^2^ = 0.992; Eff% = 98.7%), and clade IIb (*R*^2^ = 0.996; Eff% = 110.9%), respectively. These results indicate that the established multiplex qPCR method has high efficiency and strong linear correlation.

To evaluate the detection sensitivity of the multiplex qPCR, the probes of clade Ia, Ib, IIa, and IIb were labeled with FAM and ROX, respectively. The plasmids were then detected with a 10-fold gradient dilution (10^8^–10^1^ copies/μL; equivalent to 2 × 10^8^ to 2 × 10^1^ copies/reaction, as 2 μL of template was added per reaction). The results showed that the preliminary LODs of clade Ia, clade Ib, IIa, and IIb were 100, 10, 10, and 1 copies/reaction, respectively (Figures 4E–H). For clade IIb, the 1 copy/reaction LOD (equivalent to 0.5 copies/μL) was reproducible across three independent replicates, confirming its reliability as a preliminary estimate.

### 3.4 Specificity analysis of multiplex qPCR

**FIGURE 5:**
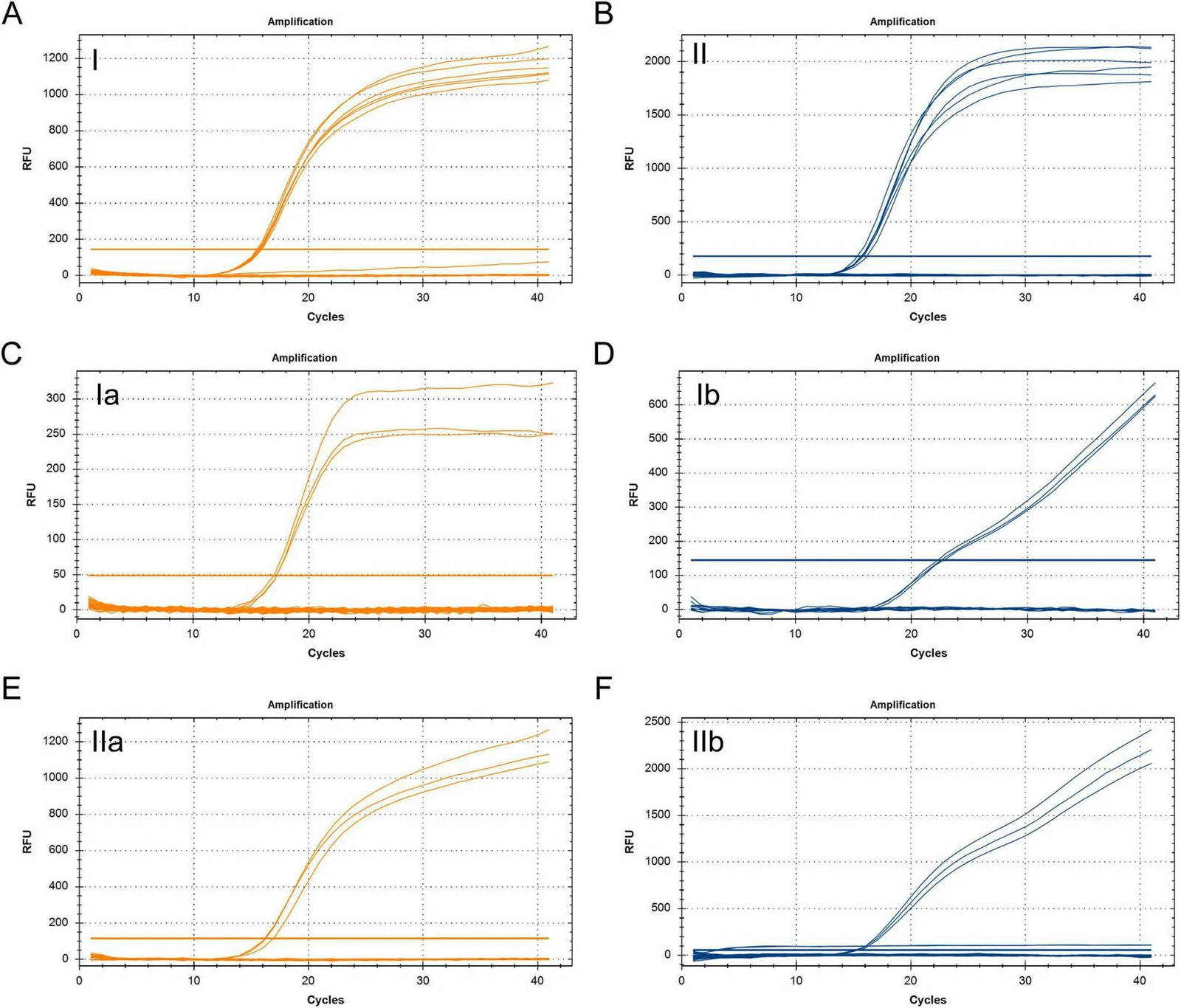
Specificity of multiplex qPCR. Fluorescent signals were observed only when MPXV clades I **(A)**, II **(B)**, Ia **(C)**, Ib **(D)**, IIa **(E)**, and IIb **(F)** were detected, but not for other viruses (VTT, HAdV, GETV, CHIKV, JEV, DENV, IAV, IBV, and SARS-CoV-2). All data represents at least three independent replicates (*n* = 3).

To evaluate the specificity of the multiplex qPCR assay, nucleic acid-positive samples of VTT, HAdV, GETV, CHIKV, JEV, DENV, IAV, IBV, or SARS-CoV-2 were used as templates for amplification with this multiplex system. The results demonstrated that only MPXV clades Ia, Ib, IIa, and IIb produced amplified signals and generated specific amplification curves, whereas no positive signals or amplification curves were observed for VTT, HAdV, GETV, CHIKV, JEV, DENV, IAV, IBV or SARS-CoV-2 (Figures 5A–F), indicating the high specificity of the developed multiplex qPCR assay.

### 3.5 Repeatability analysis of multiplex qPCR

To estimate the repeatability of the developed multiplex qPCR assay, two dilutions of plasmid standard mixtures were used to assess the intra-assay and inter-assay variation. The results showed that the CVs of Ct values of intra-assay and inter-assay ranged from 0.09 to 1.88% (Tables 3, 4) and from 0.23 to 2.68%, respectively (Tables 5, 6), indicating the excellent repeatability of the assay.

**TABLE 3:** The intra-assay coefficient of variation (CV) of the qPCR in panel 1 (*n* = 3 per condition).

	1.00E+06				1.00E+04			
	Clade Ia	Clade Ib	Clade IIa	Clade IIb	Clade Ia	Clade Ib	Clade IIa	Clade IIb
Ct values	11.94	10.47	11.81	11.56	17.55	16.36	17.25	17.84
	11.76	10.04	11.76	11.67	17.51	16.18	17.32	18.08
	11.66	10.43	11.59	11.62	17.81	16.26	17.62	18.15
Mean	11.79	10.31	11.72	11.62	17.62	16.27	17.40	18.02
SD	0.12	0.19	0.09	0.04	0.13	0.07	0.16	0.13
CV%	0.98	1.88	0.80	0.39	0.75	0.45	0.92	0.74

**TABLE 4:** The intra-assay coefficient of variation (CV) of the qPCR in panel 2 (n = 3 per condition).

	1.00E+06				1.00E+04			
	Clade Ia	Clade Ib	Clade IIa	Clade IIb	Clade Ia	Clade Ib	Clade IIa	Clade IIb
Ct values	11.11	12.5	12.2	12.71	16.78	18.27	18.48	19.01
	10.93	12.24	12.27	12.51	16.49	18.23	18.58	19.05
	10.8	12.35	12.42	12.72	16.5	18.25	18.6	19.23
Mean	10.95	12.36	12.30	12.65	16.59	18.25	18.55	19.10
SD	0.13	0.11	0.09	0.10	0.13	0.02	0.05	0.10
CV%	1.16	0.86	0.75	0.76	0.81	0.09	0.28	0.50

**TABLE 5:** The inter-assay coefficient of variation (CV) of the qPCR in panel 1 (n = 3 per condition).

	1.00E+06				1.00E+04			
	Clade Ia	Clade Ib	Clade IIa	Clade IIb	Clade Ia	Clade Ib	Clade IIa	Clade IIb
Ct values	11.66	10.04	11.59	11.29	17.51	15.45	16.28	17.17
	11.75	9.94	11.39	11.56	17.09	16.18	17.25	17.84
	11.56	9.67	11.3	11.44	17.32	15.81	16.72	17.78
Mean	11.66	9.88	11.43	11.43	17.31	15.81	16.75	17.60
SD	0.08	0.16	0.12	0.11	0.17	0.30	0.40	0.30
CV%	0.67	1.58	1.06	0.97	0.99	1.88	2.37	1.72

**TABLE 6:** The inter-assay coefficient of variation (CV) of the qPCR in panel 2 (n = 3 per condition).

	1.00E+06				1.00E+04			
	Clade Ia	Clade Ib	Clade IIa	Clade IIb	Clade Ia	Clade Ib	Clade IIa	Clade IIb
Ct values	11.11	12.05	12.42	12.51	16.19	18.23	18.48	18.57
	11.19	12.05	12.49	12.35	16.38	17.07	18.11	19.01
	11.2	12.24	12.45	12.13	16.49	17.67	17.99	18.94
Mean	11.17	12.11	12.45	12.33	16.35	17.66	18.19	18.84
SD	0.04	0.09	0.03	0.16	0.12	0.47	0.21	0.19
CV%	0.36	0.74	0.23	1.26	0.76	2.68	1.15	1.02

### 3.6 Clinical application of multiplex qPCR

The developed multiplex qPCR assay was applied to analyze the 48 nucleic acids samples collected from the skin lesions of crab-eating monkeys or SCID mice. The positive rates of MPXV clade IIb were 100% (48/48) (Table 7). However, no coinfection in any of the other three clades were observed. All 48 clinical samples were also analyzed using the singleplex qPCR assays. The results demonstrated that the concordance rates between the two methods were 100%, respectively.

**TABLE 7:** Agreement between the multiplex qPCR and singleplex qPCR.

		Multiplex qPCR		Singleplex qPCR		Agreement
		Positive	Negative	Positive	Negative	
Panel 1	Clade I	0	0	–	–	–
	Clade II	48	0	–	–	–
Panel 2	Clade Ia	0	0	–	–	–
	Clade Ib	0	0	–	–	–
	Clade IIa	0	0	–	–	–
	Clade IIb	48	0	48	0	100%

## 4 Discussion

The ongoing global circulation of multiple MPXV clades with distinct pathogenic profiles necessitates diagnostic tools capable of rapid and accurate clade discrimination. In this study, we developed and validated a two-tube multiplex qPCR assay targeting the H3L gene, demonstrating its ability to differentiate between MPXV clades Ia, Ib, IIa, and IIb with high analytical sensitivity, specificity, and reproducibility.

While several commercial and in-house MPXV qPCR assays have been described, most are designed for generic virus detection or limited lineage differentiation. For example, assays targeting F3L (GENE, 2024), N3R (Kulesh et al., 2004), or B6R (Li et al., 2006) provide excellent sensitivity for MPXV detection but cannot distinguish between clades. More recently, Fan et al. developed a multiplex assay capable of differentiating MPXV clades and sub-lineages within IIb (Fan et al., 2025); however, their approach does not encompass the Ia/Ib distinction, which has become critical following the emergence of clade Ib in 2024 (WHO, 2024c). Our assay fills this gap by providing comprehensive coverage of all four major clades in two streamlined reactions.

A distinctive feature of our assay is its two-tube, two-channel design. Because the H3L gene sequences of clades Ia, Ib, IIa, and IIb differ by only a small number of single nucleotide polymorphisms (SNPs), discriminating all four clades simultaneously in a single reaction would require at least four independent fluorescence channels—a capability not available on most clinical qPCR instruments, which typically have 2–4 channels. Our two-tube strategy circumvents this limitation by using a two-step decoding approach: Panel 1 establishes the “clade context” (clade I vs. II) using FAM and ROX channels, and Panel 2 provides the “subtype signal” (FAM or ROX) by reusing the same two channels. The final clade assignment is made by integrating results from both panels. This design enables four-clade discrimination on standard two-channel qPCR platforms, offering broader accessibility in routine clinical laboratories compared to assays requiring specialized four- or five-channel instruments. We acknowledge that this approach requires two separate reactions, which increase sample consumption and hands-on time compared to a single-tube format; however, the trade-off is justified by enhanced accessibility and the reduced risk of probe competition inherent in distributing targets across two reactions. The two-tube format reduces reagent costs and hands-on time compared to four separate singleplex reactions (Huggett et al., 2022; Mills et al., 2023).

The developed assay demonstrated excellent analytical sensitivity, with limits of detection ranging from 1 to 100 copies/reaction depending on the clade (Ia: 100 copies; Ib: 10 copies; IIa: 10 copies; IIb: 1 copy). The observed differences in LOD among clades likely reflect variations in primer/probe binding affinity, which may be influenced by the number and quality of sequences available for each clade during the design phase. Specifically, clade IIb had the largest number of high-quality sequences (68 out of 220) available for design, which may have facilitated the selection of optimally matched primer/probe sets, whereas clade Ia had relatively fewer sequences (60), potentially limiting design optimization. Importantly, even the highest LOD among the four clades (100 copies/reaction for clade Ia) is substantially lower than the typical viral loads observed in clinical skin lesion specimens (typically 10^3^–10^6^ copies/mL), indicating that the 100-fold difference among clades is unlikely to affect clinical diagnostic performance. The high amplification efficiencies (91.5–110.9%) and strong correlation coefficients (*R*^2^ > 0.990) indicate optimal reaction conditions. The assay’s specificity was confirmed by the absence of cross-reactivity with nine other human pathogens, including the closely related orthopoxvirus VTT and clinically relevant viruses that may present similar symptoms (e.g., HAdV, DENV, SARS-CoV-2).

The low intra- and inter-assay coefficients of variation (CVs < 2.7%) demonstrate robust reproducibility, essential for consistent performance across different runs and laboratories. The total assay time of approximately one hour enables same-day result reporting, facilitating timely clinical decision-making and outbreak response.

Validation using 48 specimens from infected non-human primates (crab-eating macaques and SCID mice), all confirmed as clade IIb, showed 100% concordance with singleplex qPCR for clade IIb detection. We acknowledge several important limitations. First, the 48 specimens were derived from animal models rather than human patients; therefore, our results represent preclinical validation, and formal clinical validation in human specimens is required. Second, clade assignment of the specimens was not confirmed by sequencing; sequencing remains the gold standard for clade discrimination, and its absence represents a limitation. Third, clinical specimens representing clades Ia, Ib, and IIa were unavailable because our laboratory is located in a non-endemic region; their performance was therefore validated only analytically using plasmid standards. We have explicitly called for future clinical evaluation in endemic regions where these clades are circulating.

We acknowledge that the performance of our assay on mixed templates containing two or more MPXV clades has not been experimentally verified, and we recognize this as an analytical limitation of the current study. We do not intend to dismiss this limitation solely based on the rarity of mixed infections; rather, we provide the following considerations:

First, the high reproducibility of our assay on single-template specimens (intra-assay CV: 0.09–1.88%; inter-assay CV: 0.23–2.68%) and the consistent amplification efficiencies across all four clades (91.5–110.9%) suggest that the primer/probe systems are robust and well-optimized, which would minimize the risk of probe competition in mixed-template scenarios.

Second, we acknowledge that, due to the two-channel design, in the event of a mixed infection involving specific cross-clade combinations (e.g., clades Ia + IIb versus Ib + IIa), the assay would produce indistinguishable signal patterns and cannot resolve the specific pairing of subtypes. This is not a technical flaw, but an inherent limitation of the two-channel multiplexing strategy: Panel 2 reuses FAM to detect both Ia and IIa, and ROX to detect both Ib and IIb. Without a third or fourth fluorescence channel, resolving such cross-clade mixtures is fundamentally impossible. We explicitly note that this limitation applies only to the exceptionally rare scenario of cross-clade mixed infections.

Third, according to surveillance data from the ongoing global mpox outbreak, among more than 170,000 confirmed cases reported across 133 member states as of December 31, 2025, no MPXV clade I/II co-infections have been documented (Bunge et al., 2022). While co-circulation of different clades in the same geographic region has been reported, actual co-infection with distinct MPXV clades in a single patient remains exceptionally rare. Therefore, while we agree that mixed-template characterization would strengthen the assay’s analytical validation, the practical clinical impact of this limitation is minimal.

We have explicitly listed mixed-template validation—using artificially mixed plasmid standards and, if available, clinical specimens—as a priority for future work, to fully characterize this analytical limitation.

Regarding cross-reactivity validation, we acknowledge that a more comprehensive panel of orthopoxviruses (e.g., cowpox virus, camelpox virus, ectromelia virus) would further strengthen the specificity data. However, these samples were not available in our laboratory due to restricted access and biosafety requirements in China. Importantly, among all orthopoxviruses, vaccinia virus (VACV) is phylogenetically the closest relative to MPXV; the absence of cross-reactivity with the VACV Tiantan strain—the most challenging cross-reactivity candidate—provides strong evidence that the assay is unlikely to cross-react with other, more distantly related orthopoxviruses. Regarding host genomic DNA interference, we note that all 48 specimens used in this study were derived from skin lesions and thus contained host genomic DNA. The successful detection of MPXV in all 48 specimens empirically demonstrates that host genomic DNA background does not significantly interfere with assay performance. Given the high genomic similarity between macaque and human genomes, this finding is likely to extrapolate to human clinical specimens, particularly for skin lesion specimens—the WHO-preferred sample type for mpox diagnosis—which typically contain abundant viral DNA relative to host nucleic acids.

We acknowledge that our LOD determination using three replicates by 10-fold serial dilution represents a preliminary estimate and does not fully conform to CLSI EP17-A2 or MIQE guidelines for formal LOD validation. While this provides a reliable initial assessment of analytical sensitivity, we recognize that formal LOD validation with ≥ 20 replicates per concentration and finer concentration gradients would be required for regulatory approval, and we plan to conduct such validation in future work. Importantly, even the highest preliminary LOD among the four clades (100 copies/reaction for clade Ia) is substantially lower than typical viral loads observed in clinical skin lesion specimens (10^3^–10^6^ copies/mL), indicating that the 100-fold difference among clades is unlikely to affect clinical diagnostic performance. All LOD values have been uniformly reported as copies/reaction (with 2 μL template per reaction) to ensure clarity. Future work should include: (i) prospective clinical evaluation in mpox-endemic settings where multiple clades co-circulate, with sequencing confirmation; (ii) formal LOD validation according to CLSI guidelines; (iii) adaptation to point-of-care platforms for decentralized testing (Yigci et al., 2025); and (iv) integration with sequencing-based surveillance to monitor for emerging variants that may affect primer/probe binding.

The ability to rapidly distinguish between MPXV clades has direct implications for public health response. During the 2024 clade Ib outbreak, early discrimination from endemic clade Ia cases was crucial for understanding transmission dynamics and implementing targeted interventions. Our assay provides a practical tool for front-line laboratories to generate this critical information without requiring sequencing capacity, using standard two-channel qPCR instruments widely available in clinical settings.

In summary, this multiplex qPCR method was developed based on bioinformatics analyses of 220 MPXV genomic sequences encompassing all four clades from public databases. The method exhibits high specificity, demonstrating no cross-reaction with other human pathogens tested. It has high sensitivity, with limits of detection ranging from 1 to 100 copies/reaction. The qPCR assay exhibited remarkable repeatability, with CV values below 2.7%. The developed method can complete the detection of MPXV and the differentiation of its clade within 1 hour. The findings reveal that the two-tube multiplex qPCR method is effective in detecting MPXV clades Ia, Ib, IIa, and IIb rapidly and accurately in analytical settings. The ability to distinguish between these clades provides a great advantage for epidemiological studies and epidemic management, although further clinical validation is needed before routine clinical implementation for all four clades.

## 5 Conclusion

In conclusion, we have developed a two-tube multiplex qPCR assay that enables rapid, sensitive, and specific differentiation of MPXV clades Ia, Ib, IIa, and IIb in analytical settings. With excellent analytical performance, robust reproducibility, and promising preclinical applicability demonstrated for clade IIb in animal-derived specimens, this assay represents a valuable addition to the diagnostic armamentarium for mpox. Its capacity to discriminate emerging clades using standard two-channel qPCR instruments positions as a useful tool for clinical management, epidemiological surveillance, and future outbreak preparedness. However, we emphasize that the current validation is primarily analytical; formal clinical validation with diverse human clinical specimens from clades Ia, Ib, and IIa, particularly from endemic regions with sequencing confirmation, is warranted before the assay can be recommended for routine clinical use for all four clades.

## Data Availability

The original contributions presented in the study are included in the article/supplementary material, further inquiries can be directed to the corresponding author.

## Footnotes

1
https://www.gisaid.org/

2
https://www.ncbi.nlm.nih.gov/nucleotide/
